# Measurement and correlates of empathy among female Japanese physicians

**DOI:** 10.1186/1472-6920-12-48

**Published:** 2012-06-22

**Authors:** Hitomi U Kataoka, Norio Koide, Mohammadreza Hojat, Joseph S Gonnella

**Affiliations:** 1 Department of Primary Care and Medical Education, Okayama University Medical School, Okayama, Japan; 2 Department of General Medicine, Okayama University Medical School, Okayama, Japan; 3 Center for Research in Medical Education and Health Care, Jefferson Medical College of Thomas Jefferson University, Philadelphia, USA

**Keywords:** Empathy, Female physicians, Career development

## Abstract

**Background:**

The measurement of empathy is important in the assessment of physician competence and patient outcomes. The prevailing view is that female physicians have higher empathy scores compared with male physicians. In Japan, the number of female physicians has increased rapidly in the past ten years. In this study, we focused on female Japanese physicians and addressed factors that were associated with their empathic engagement in patient care.

**Methods:**

The Jefferson Scale of Empathy (JSE) was translated into Japanese by using the back-translation procedure, and was administered to 285 female Japanese physicians. We designed this study to examine the psychometrics of the JSE and group differences among female Japanese physicians.

**Results:**

The item-total score correlations of the JSE were all positive and statistically significant, ranging from .20 to .54, with a median of .41. The Cronbach’s coefficient alpha was .81. Female physicians who were practicing in “people-oriented” specialties obtained a significantly higher mean empathy score than their counterparts in “procedure-” or “technology-oriented” specialties. In addition, physicians who reported living with their parents in an extended family or living close to their parents, scored higher on the JSE than those who were living alone or in a nuclear family.

**Conclusions:**

Our results provide support for the measurement property and reliability of the JSE in a sample of female Japanese physicians. The observed group differences associated with specialties and living arrangement may have implications for sustaining empathy. In addition, recognizing these factors that reinforce physicians’ empathy may help physicians to avoid career burnout.

## Background

Empathy is essential for achieving optimal outcomes in patient care. Although the importance of empathy has been recognized and emphasized, the concept of empathy has a long history marked by ambiguity in its definition and measurement. Hojat and colleagues at Jefferson Medical College proposed the following definition of empathy in the context of patient care: “Empathy is a predominantly *cognitive* (rather than emotional) attribute that involves an *understanding* (rather than feeling) of experiences, concerns and perspectives of the patient, combined with a capacity to *communicate* this understanding and an *intention to help*.” [1,2]. Hojat et al. developed and validated the Jefferson Scale of Empathy (JSE) to measure empathy specifically in medical students and physicians in the context of patient care [2-6]. Using the JSE, various interesting findings have been reported. For example, in an analysis of empathy scores with respect to gender and specialty, females consistently scored higher than males [5,6]. After controlling for gender, psychiatrists and generalist physicians scored significantly higher than did physicians specializing in “technology-oriented specialties” such as anesthesiology, orthopedic surgery, neurosurgery, radiology, cardiovascular surgery, obstetrics–gynecology, and general surgery [5].

In Japan, females constitute approximately 30% of all medical students, and the number of female students has increased rapidly in the past decade. In spite of the increasing number of women in medicine, one study reported a marked decline in workforce participation, especially among female physicians in their late 20s and 30s [7]. This is likely due to the conflicting work and family schedules that female physicians experience. In Japan, female physicians are reported to work fewer hours than male physicians and to be more frequently in part-time practice, similar to reports from other countries. However, various approaches are available to improve the recruitment and retention of female physicians by providing a balance for work/family life [8]. It would be desirable to understand female physicians’ concerns for improving this balance so that they can more easily continue practicing medicine and serving patients. In this study, we focused on female Japanese physicians, and addressed the factors that were associated with their empathic engagement in patient care.

## Methods

### Participants

Of the 1,364 female physicians who graduated from Okayama University Medical School or worked at affiliate hospitals of Okayama University Hospital, 285 volunteers (21%) returned a completed JSE in 2008.

### Instrument

The physician version (HP-Version) of the JSE used in this study includes 20 items answered on a 7-point Likert-type scale (1 = strongly disagree, 7 = strongly agree). Satisfactory evidence in support of the psychometric properties of this scale has been reported [2-6]. The JSE has been receiving international attention from researchers, and has been translated into 42 languages and used in 60 countries worldwide including those in Europe, the Middle East, Africa, Asia, North America, South America, and in New Zealand [9].

### Procedures

The JSE was first translated into Japanese by one of the authors (HUK) by using a back-translation procedure [10]. In 2008, we distributed the translated version of the JSE to 1,364 female physicians (age range from 24 to 77 years) who had graduated from Okayama University Medical School, or worked at affiliate hospitals of Okayama University Hospital. We explained that the instrument was about empathy and that we would use the results for research purposes. The study was approved by the university’s research ethics committee. Physicians were not compensated for their participation in the study.

### Statistical analyses

We calculated the Pearson correlation coefficients to examine the item-total score correlations. The item-total score correlations were calculated based on responses to each item and the total score of the JSE, minus the corresponding item. We calculated the Cronbach co-efficient alpha to assess the internal consistency aspect of reliability of the instrument. We used t-test and analysis of variance to test the significance of differnces in group comparisons.

## Results

### Response rate

The group of respondents included in this study comprised 21% of the population of female physicians in the area. Although the response rate was low, the age range from 24 to 77 covered a broad sample representation. No substantial differences were observed for the physicians’ ages and specialties between those who responded to the survey and those who did not.

### Descriptive statistics at the item level

Examination of the responses to different items revealed that all of the points on the 7-point scale were used by respondents, with the exception of one item for which the response range was 2–7. The item mean scores ranged from a low of 4.0 (on a 7-point scale) for “because people are different, it is difficult for me to see things from my patients’ perspectives” (a reverse score item), to a high of 6.3 for “I believe that empathy is an important therapeutic factor in medical and surgical treatment.” The item standard deviations ranged from .93 to 1.6. The item-total score correlations ranged from .20 for “I do not enjoy reading non-medical literature or the arts” (a reverse scored item) to a high of .54 for “I try to understand what is going on in my patients’ minds by paying attention to their non-verbal cues and body language.” The median item-total score correlation was .41, and the overall item-total score correlations were statistically significant (*p* < .01).

### Descriptive statistics of the scale

An examination of the score distribution revealed a bell-shape, with a mean of 110.4, standard deviation of 11.9, and a median of 110. The 25^th^ and 75^th^ percentiles were 103 and 119, respectively. The skewness index was near zero (−.21), indicating that the distribution was symmetric and the kurtosis index was closed to zero (−.06), indicating that the score distribution was mesokurtic, or a bell-shape normal distribution. The score for the entire sample ranged from 76 to 137 (possible range 20–140). The descriptive statistics and reliability coefficient of the scale are reported in Table 1.

**Table 1:** Descriptive Statistics for the Jefferson Scale of Empathy Administered to 285 Female Japanese Physicians

**Statistics**	**Value**
Mean	110.4
Standard Deviation	11.9
25^th^ Percentile	103
50^th^ Percentile (Median)	110
75^th^ Percentile	119
Possible Range	20 – 140
Actual Range	76 – 137
Skewness	-.21
Kurtosis	−06
Reliability (Cronbach’s coefficient alpha)	.81

### Reliability

The Cronbach coefficient alpha, an indicator of the internal consistency reliability of the measuring instrument, was .81, which is in an acceptable range for psychological measures. A similar reliability coefficient was reported for American medical students (*r =* .80)[1], American physicians (*r* = .80) [6], and Japanese medical students (*r =* .80) [10].

### Comparison of scores for physicians in different specialties

The mean scores and standard deviations of empathy for female physicians by specialties are reported in Table 2. As shown in the table, the mean empathy score of physicians in people-oriented specialties (*e.g.*, general internal medicine, general pediatrics, and psychiatry) was significantly higher than those in technology-oriented specialties (*e.g.*, anesthesiology, surgery, pathology, radiology, ophthalmology, orthopedic surgery, urology, and obstetrics/gynecology). The mean scores for physicians in people-oriented specialties and technology-oriented specialties were 112.9 and 106.9, respectively. The difference was statistically significant (*F*_(2,282)_ = 8.4, *p* < .001). The mean empathy score for physicians in other specialties (emergency medicine, public health, rehabilitation medicine) was not significantly different from the two aforementioned groups.

**Table 2:** A Comparison of the Scores on the Jefferson Scale of Empathy Among 285 Female Japanese Physicians in Different Specialties

**Specialties**	***n***	**Mean**	**SD**
People-oriented^1^	156	112.9	11.7
Technology-oriented^2^	98	106.9	11.2
Other^3^	31	108.4	13.1

*F*_(2,282)_ = 8.4, *p* < .001 (people-oriented > technology-oriented = other)

^1^ People-oriented specialties included general internal medicine, general pediatrics and psychiatry.

^2^ Technology-oriented specialties included anesthesiology, surgery and surgical specialties, pathology, radiology, ophthalmology, orthopedics, obstetrics/gynecology, and urology.

^3^ Other specialties included emergency medicine, public health, rehabilitation medicine, and unspecified specialties.

### Comparison of scores based on different living arrangements

The mean scores and standard deviations of empathy for female physicians by different living arrangements are reported in Table 3. As shown in the table, the physicians living with their parents in an extended family or living close to their parents, (*M* = 113.0, *SD* = 12.1) outscored physicians living alone or living in a nuclear family (which includes only spouses without parents or other family members) (*M* = 109.6, *SD* = 11.8). The diffe-rence was statistically significant (*t*_(256)_ = 1.99, *p* < .05).

**Table 3:** A Comparison of the Scores on the Jefferson Scale of Empathy Among 285 Female Japanese Physicians by Different Living Arrangements

**Living Arrangements**	***n***	**M**	**SD**
Living with parents	69	113.0	12.1
Living alone, or nuclear family	189	109.6	11.8

*t*_(256)_ = 1.99, *p* < .05. (27 physicians did not respond to the living arrangement question).

We also examined the associations between empathy scores, age and years of clinical experiences. The correlation between physicians’ empathy scores and age was positive but negligible (*r* = .11, *p* = .07), and the correlation between empathy scores and years of practicing medicine was statistically significant but low in magnitude (*r* = .15, *p* < .05).

## Discussion

The findings of this study supported different aspects of the psychometrics of the Japanese translation of the JSE. For example, the method using contrasted groups [11] showed that there were significant differences between physicians in people-oriented and technology-oriented specialties. This also confirmed the validity of the Japanese version of the JSE, because this finding was in the expected direction, consistent with findings previously reported in the literature. The significant item-total score correlations indicated that each single item contributed significantly to the total score. The internal consistency reliability of the Japanese scale was supported by the Cronbach’s coefficient alpha.

Female medical students and physicians have previously been reported to score higher than their male counterparts [2-6,10]. In Japanese medical students, for example, we noticed that the mean scores of the JSE were 103.7 and 107 for men and women, resprctively [10]. The gender difference in empathy has been attributed to intrinsic factors (*e.g.*, evolutionary-biological gender characteristics) as well as extrinsic factors (*e.g.*, socialization, gender role expectations, *etc.*) [2,5]. In the present study, we further analyzed the factors that contribute to variations in female physicians’ empathy scores.

To accomplish this analysis, we first examined the differences in empathy scores between female physicians in different specialties. Those in people-oriented specialties outscored those in technology-oriented specialties. Consistent with our findings, several previous studies reported that people-oriented specialists had higher empathy scores [5,6,12]. Medical students who pursued their training in people-oriented specialties have been reported to score higher in empathy in all years of medical school [1]. Our previous study also revealed that female students outscored their male counterparts in medical school, and proportionally more female students chose people-oriented specialties [10]. In Japan, students can freely choose their specialties, and female medical students are more interested in choosing internal medicine and pediatrics as their specialties [13]. This trend was expected that female students outscored in empathy and choose people-oriented specialties. Our study group comprised 30.9% internists, which could have contributed to the higher empathy score reported by physicians in people-oriented specialties in this study. However, if female physicians practicing in people-oriented specialties experience a decline in empathy because of difficulties in balancing work and life and unable to continue their career; this may present problems for a society in which there is a shortage of physicians in this important area [14].

Consistent with previous findings [2,6], we noticed that empathy score was higher in physicians who were practicing in people-oriented specialties (*e.g.*, general internal medicine). This implies that empathy can be better manifested and maintained in specialties that require continuous care. This speculation is justified considering that people-oriented specialties often require first encounter and continuous care that is likely to lead to an empathic engagement in a long-term patient-physician relationship.

Rotor et al. reported that female physicians engaged in significantly more active partnership behaviors, positive talk, psychosocial counseling, psychosocial question asking, and emotionally focused talk [15]. These features of communication seen in female physicians are important aspects of empathy. In addition, if physicians have more variety of experiences in their life, their attitude and understanding for patients’ life could be more insightful. From this standpoint, it is advantageous for female physicians who are juggling their work and personal life to pursue people-oriented specialties. These experiences could ensure the enhancement of communication that would strengthen their empathic engagement in patient care.

We investigated the factors that could contribute to the variations in empathy scores among female physicians such as the work place, working style, living arrangements, job satisfaction, experience of leaving the job, marital status, and having children. Among these factors, only the living arrangement had a significant association with empathy scores. Female physicians in Japan traditionally perform their social roles as mothers or home makers in addition to their professional responsibilities as physicians. In Japan, mothers are expected to be the primary care provider for their children. Because the number of employed mothers is increasing in Japan, the number of childcare centers and kindergartens is insufficient, and there are many children on waiting lists to enter these facilities. In such situations, female physicians who have children face difficulties juggling their career and raising children. However, when they live with their parents or extended family, they may benefit from increased family support. When female physicians have optimal family support, they are likely to continue their career with less stress and exhaustion. It is to note that the empathy of physicians with no children who were living with or close to their parents (*M* =111.0), and for those with children who were living in a nuclear family (*M =*110.06) was similar. A previous study revealed that having children decreased female physicians’ stress [16,17]. Our study suggested that the presence of “significant others” would increase the empathy of female physicians. Although we did not ask about close friends and supporters of female physicians, such relationships may influence their empathy.

Female physicians are often married to male physicians in Japan, and this same occurrence also been reported in other countries [18,19]. In our survey, more than 70% of married female physicians reported having physician husbands (data not shown). In such relationships, the pressure and work demands would be greater, and controlling their lifestyle and workload would be more difficult than for couples where the spouse had a less demanding career. On the other hand, one can argue that marrying to a male physician could be beneficial to female physicians because they would have more support and understanding from the spouse. In the physician work-life study, female physicians were reported to have 1.6 times the odds of experiencing burnout compared with males (*P* < .05), with the odds of burnout by the female physicians increasing by 12% to 15% for each additional five hours worked per week over 40 hours (*P* < .05)[16]. Excessive stress, sleep deprivation, and a demanding lifestyle can increase the risk of burnout, leading to erosion of empathy in physicians. In the same report, the authors suggested that for female physicians with young children, the odds of burnout decreased 40% when they were supported by colleagues, a spouse, or significant others to help them balance career and home responsibilities [16]. Taking on multiple roles as a physician, wife, and mother is challenging for female physicians. However, our study suggests that living with extended family could contribute to maintaining high empathy, regardless of the marital status of physicians. This could be a key factor that may help to avoid the risk of burnout and exhaustion in female physicians. At the same time, the understanding of their surrounding people such as husbands, and colleagues is important. In addition, better work based child care facilities would be beneficial to female physicians to maintain their clinical career.

Empathy is essential for achieving optimal outcomes in patient care. Our findings suggest that there are several factors, such as the choice of specialty and living arrangements, that can mitigate the risk of erosion of empathy in the practice of medicine among female physicians in Japan.

## Conclusions

This is the first study that analyzed Japanese female physicians’ empathy using the JSE. The present results support the validity and reliability of the Japanese translation of the JSE. In addition, consistent with the previous findings, the female physicians in our study who were practicing in “people-oriented” specialties had significantly higher mean empathy scores than did their female counterparts in “procedure-or technology-oriented” specialties.Physicians who reported living with their parents in an extended family scored higher on the JSE than those who were living alone or in a nuclear family, suggesting that physicians who benefit from family support can better maintain their empathy.

It should be noticed that this is a correlational study, and the findings do not by any means suggest any cause and effect relationship. Further research is needed to examine the behavioral manifestation of physician empathy, rather than self-reported empathy used in the present study, although strong evidence is available tosupport the validity of the JSE [2-6,9,10], and particularly its predictive validity in patient outcomes [20]. Further research is also needed with a more representative sample of Japanese physicians to generalize our findings, and to identify other critical factors that contribute to female physicians leaving medical practice in Japan.

## Competing interests

The authors declare that they have no competing interests.

## Authors’ contributions

HKU, MH and JSG conceived the project. HKU and NK collected the data. HKU and MH conducted the statistical analyses. All authors interpreted the results, drafted the manuscript and read and approved the final manuscript.

## Pre-publication history

The pre-publication history for this paper can be accessed here:

http://www.biomedcentral.com/1472-6920/12/48/prepub
